# Supplementary material for: Synthesizing topological structures containing RNA
Source: Nat Commun. 2017 Mar 31;8:14936. doi: 10.1038/ncomms14936 (PMC5381007; doi:10.1038/ncomms14936)
Supplement: Supplementary Information — Supplementary Figures, Supplementary Tables and Supplementary References. [file ncomms14936-s1.pdf]

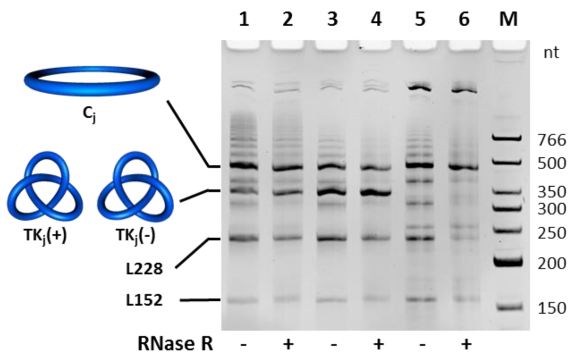

Supplementary Figure 1 | Construction of the ssRNA trefoil knots of both handednesses. The crude ligation mixtures were analyzed by dPAGE. Lanes 1 and 2 contain the sample for the right-handed knot, **TK$_j$(+)**; lanes 3 and 4 contain the sample for the left-handed knot, **TK$_j$(-)**; lanes 5 and 6 contain the control sample without staples and **C$_j$** is the generated. In lanes 2, 4 and 6, the samples were treated by RNase R, which should digest all the linear RNA species. We found that the RNase R is inhibited, especially for samples in lanes 2 and 4. This is probably due to the presence of DNA species in the sample. The yield of synthesis was estimated by the fluorescence intensity of the bands in gel and the yield for **TK$_j$(+)** and **TK$_j$(-)** is 13% and 28%, respectively. As a comparison, in our previous construction of 210-nt ssDNA knots, the yield for the positive and negative knots is 14% and 29%, respectively[1]. Linear species with length of multiples of 76 nt are major byproducts of the ligation. Two of them (**L152** and **L228**) are denoted and the other longer ones can be readily identified by comparing to the mobility of the size markers. The linear reference, **L$_j$**, was purified as the byproduct (**L228**) in all the ligation reactions, and therefore it is a mixture of linear ligated product with different connectivity.

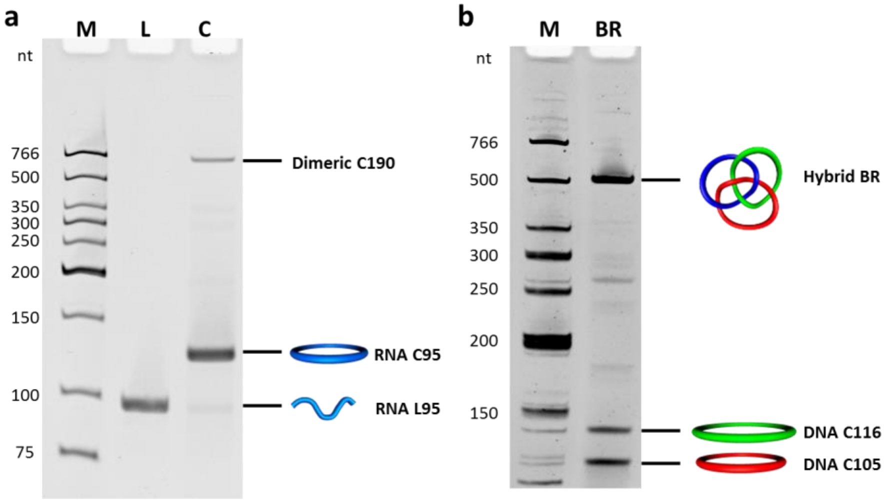

Supplementary Figure 2 | Construction of the hybrid BR. a, Preparation of the 95-nt ssRNA ring. Lane L is the linear precursor, lane C is the ligated product, which is used for the preparation of the hybrid BR. **b**, dPAGE analysis of crude ligation product for the hybrid BR. The yield was estimated to be 48% based on the conversion of the 105-nt ssDNA ring (red). Its yield is slightly lower than the previously constructed ssDNA BR, which is 55%. This is probably due to the RNA cleavage during the preparation.

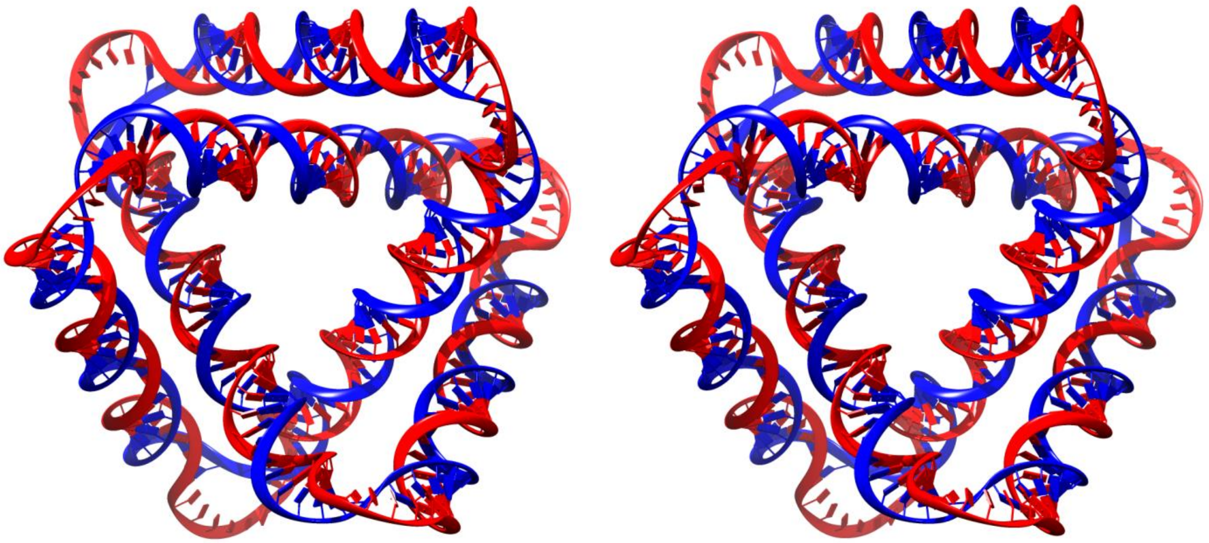

Supplementary Figure 3 | Stereo views for the designed ds RNA-DNA hybrid knots. The DNA template strand is in red and complementary RNA strand is in blue. The model and image are prepared and generated with the software Chimera.

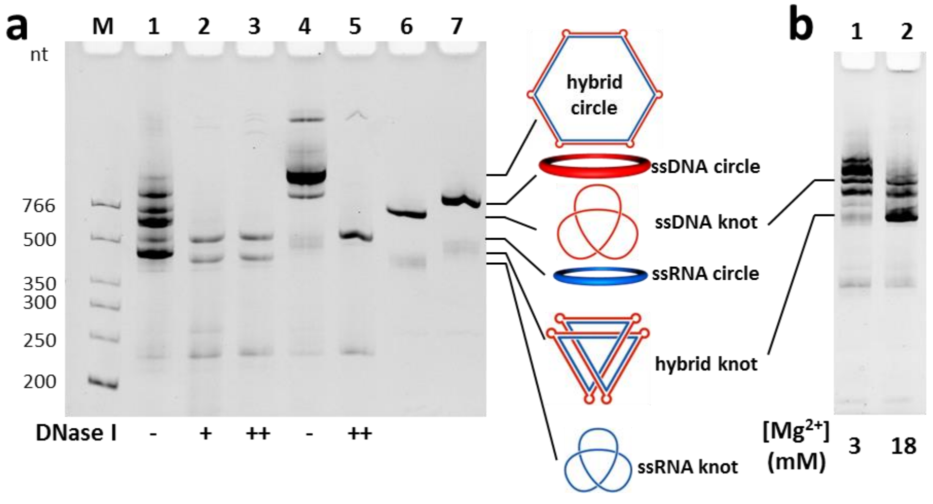

Supplementary Figure 4 | Construction of the ds RNA-DNA hybrid trefoil knot. a, dPAGE analyses of the crude products of the ds RNA-DNA hybrid trefoil knot (lanes 1 to 3) and the corresponding circle (lanes 4 and 5). The samples in lanes 2, 3 and 4 were digested by DNase I (+ or ++ means 0.04 or 0.2 unit of DNase I per µL of reaction) to confirm the topology. This was a pilot preparation with 10 mM of Mg^{2+} during the annealing. The yield for the ds hybrid knot and circle are 41% and 85%, respectively. **b**, Optimizing the yield of ds hybrid knot by adjusting the concentration of Mg^{2+} during the annealing. In the presence of 3 mM Mg^{2+}, there was almost no desired product generated (lane 1). Increasing the Mg^{2+} concentration to 18 mM increases the yield for desired product to 54%. In the large-scale preparation of ds hybrid knot, 18 mM of Mg^{2+} was used. Higher concentration of Mg^{2+} may shield the charge repulsion between the negatively charged ds RNA-DNA duplex in the compact structure or alter the dynamic conformation of the RNA-DNA hybrid duplex. The byproducts in the preparation of ds hybrid

knot are the different hybridized complexes formed by ssDNA knot and ssRNA circle. The Mg^{2+} concentration (from 3 to 18 mM) has almost no effect on the yield of the ds hybrid circle (data not shown).

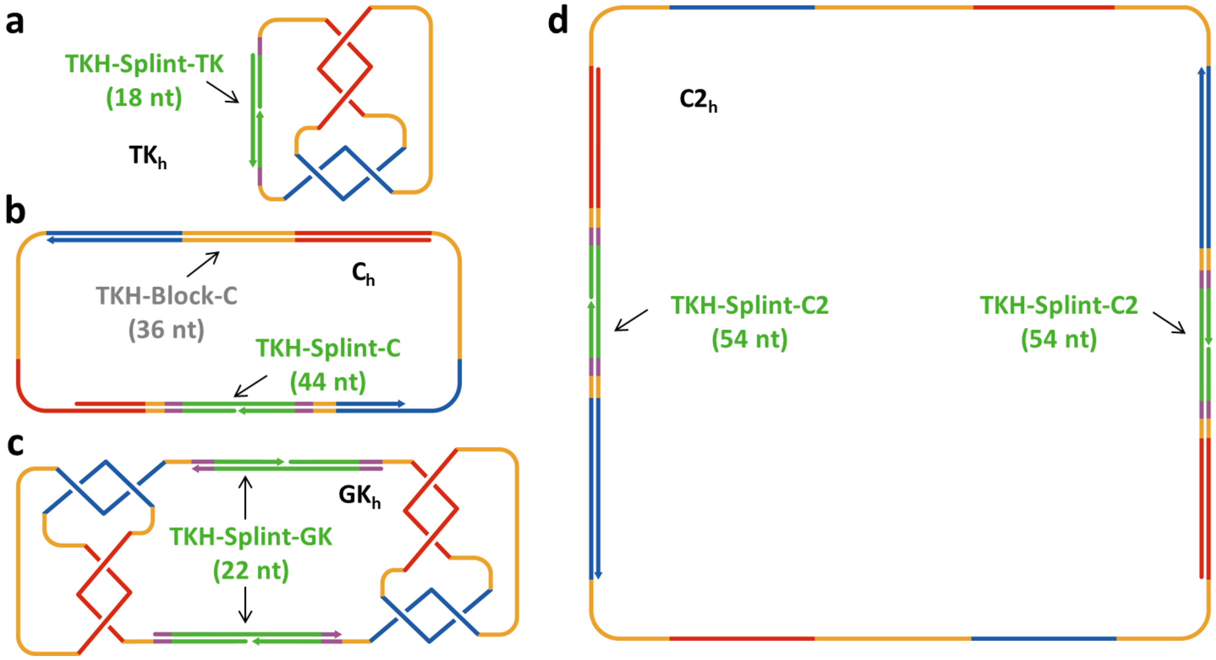

Supplementary Figure 5 | Schematics illustrating the preparation of four different helix-based topological structures using the same linear RNA precursor. a, Preparation of the monomeric trefoil knot (**TK$_h$**) via Seeman's helix-based method[2]. Different regions in the sequence are in different colors in accordance to **Supplementary Table 5**. **b**, Preparation of the monomeric circle (**C$_h$**). **C$_h$** is the trickiest structure to construct in all these four structures. On the one hand, the intrastrand base-pairing should be inhibited. On the other hand, the single-stranded linker should be sufficiently long for the cyclization of the monomer. Therefore, a 36-nt block strand (TKH-Block-C) and a 44-nt splint (TKH-Splint-C) are used to inhibit the formation of the two helical region in the RNA. **c**, Preparation of the dimeric granny knot (**GK$_h$**). A 22-nt splint (TKH-Splint-GK), without affecting the formation of helices in the RNA, decreased the single-stranded linker regions and therefore the dimeric knot is formed. **d**, Preparation of the dimeric circle (**C2$_h$**). The 54-nt splint (TKH-Splint-C2) is long enough to inhibit the formation of helices in the RNA and to discourage the cyclization of the monomer.

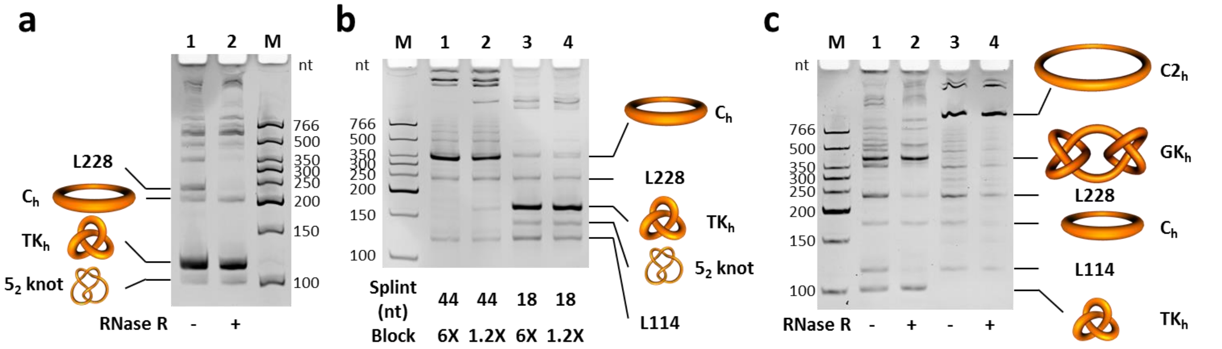

Supplementary Figure 6 | Construction of four different helix-based topological structures using the same linear RNA precursor. **a**, Preparation of the monomeric trefoil knot (**TK$_h$**). Lane 2 contains the RNase R treated sample. Note that the linear precursor (**L$_h$**) and **TK$_h$** have the same mobility under this dPAGE (8%) condition. A minor product migrating fast than **TK$_h$** is a more complex knot (probably a 5_2 knot[3]), which reflects the undesired braiding of the single-stranded linker region of the helix-based method. **b**, In order to prepare the monomeric circle (**C$_h$**), both the 36-nt block strand (TKH-Block-C) and the 44-nt splint (TKH-Splint-C) are necessary to inhibit the base-pairings of RNA and generate **C$_h$** (lanes 1 and 2). However, the combination of the 36-nt block strand and the 18-nt splint (TKH-Splint-TK) used in the preparation of **TK$_h$** only generated the **TK$_h$** (lanes 3 and 4). Changing the stoichiometry of the block strand has very small effect on the synthesis. In lanes 1 and 3, 6 equivalents of block strand to RNA was used; in lanes 2 and 4, 1.2 equivalents of block strand was used. 6 equivalents of block strand were used in the large-scale preparation due to the slightly increased yield (compare lanes 1 and 2). **c**, Preparation of the dimeric granny knot (**GK$_h$**) (lanes 1 and 2) and the dimeric circle (**C2$_h$**) (lanes 3 and 4). Lanes 2 and 4 contain the RNase R treated samples. These four helix-based structures were prepared solely to be compared with the junction-based knot as the RNA topoisomerase probe and the preparation methods (as illustrated in **Supplementary Figure 5**) of them were not optimized. Note that the electrophoretic mobility of the circular and knotted species relative to the linear species and the size markers is different in different concentration of gels (8% gel in **a**, 10% in **b**, and 7% in **c**). This is because the different topological structures have different slopes and intercepts in the Ferguson plots[3], in which the logarithm of the electrophoretic mobility (M) is plotted as a function of the total acrylamide concentration (T): $\log(M) = \log(M_0) - K_R T$[4].

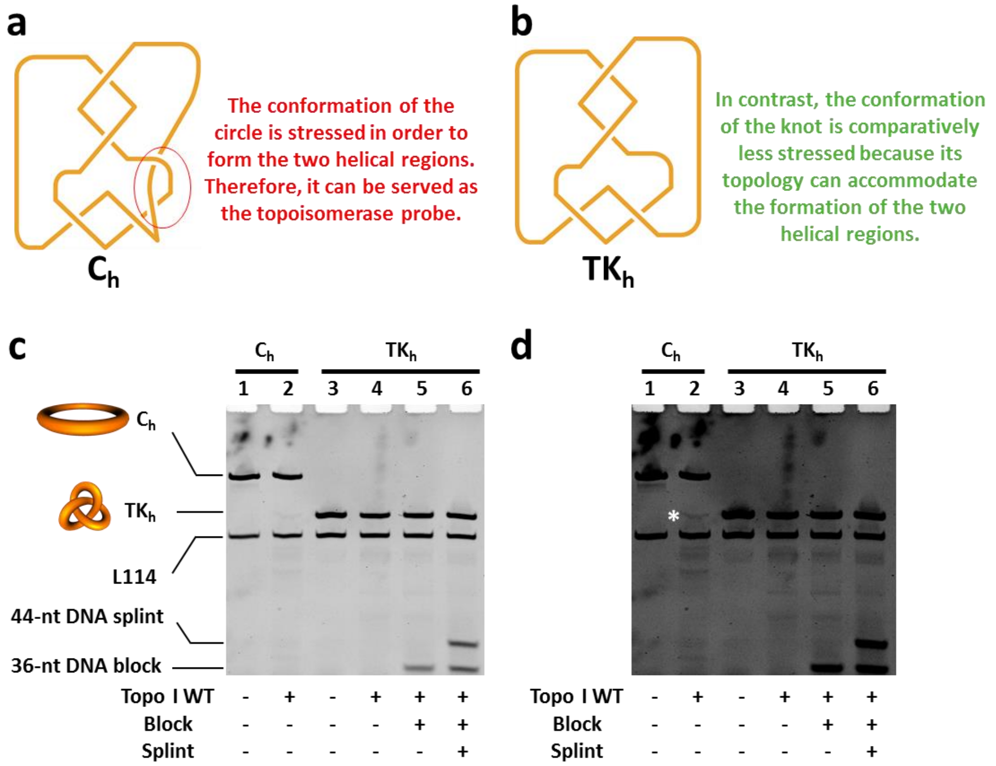

Supplementary Figure 7 | As for the helix-based topological structures, the circle $\mathbf{C_h}$, but not the trefoil knot $\mathbf{TK_h}$, should be used as the RNA topoisomerase probe. a, $\mathbf{C_h}$ is the stressed topological structure because the topology of the circle cannot accommodate the formation of the two 2-turn helices without forming extra crossovers (or nodes) as highlighted by the red oval. **b**, In contrast, the topology of the trefoil knot $\mathbf{TK_h}$ can accommodate the formation of the two 2-turn helical regions. Note that the number of nodes is expected to be 6 and 4 for $\mathbf{C_h}$ and $\mathbf{TK_h}$, respectively, in the native condition, rather than 0 and 3 (their respective minimum numbers of nodes). **c**, In the presence of Topo I, very small portion of $\mathbf{C_h}$ is converted to $\mathbf{TK_h}$ (lane 2). However, there is no detectable conversion from $\mathbf{TK_h}$ to $\mathbf{C_h}$ (lane 4), even with the addition of the 36-nt DNA block strand (lane 5) or with both the 36-nt DNA block strand and 44-nt splint strand (lane 6), both of which are used in the preparation of $\mathbf{C_h}$ to inhibit the formation of intramolecular base-pairings as shown in **Supplementary Figure 5b**. In the assays, 160 nM of RNA substrates were treated by 640 nM of wild type Topo I at 37 ℃ for 30 min. If added, the concentration of assisting DNA was 160 nM. **d**, The same gel image of **c** presented with a different contrast level to show the very faint band of product $\mathbf{TK_h}$ in lane 2 (denoted by a white asterisk).

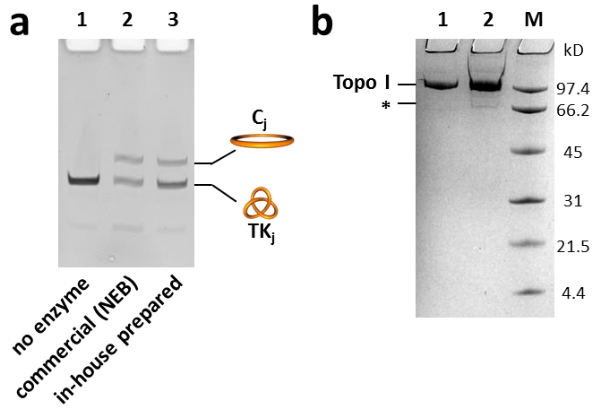

Supplementary Figure 8 | Comparing the commercial and the in-house prepared Topo I. a, the RNA topoisomerase activity for commercial product of *E. coli* topoisomerase I (NEB) (lane 1) is slightly higher than the in-house prepared enzyme (lane 2). **b**, There is possible contamination of *E. coli* topoisomerase III (73 kD) in NEB's product of *E. coli* topoisomerase I (97 kD) as analyzed by SDS-PAGE (Coomassie blue stained). Lanes 1 and 2 were loaded with 2 and 6 µg of protein, respectively. In lane 2, there is an impurity band (denoted by an asterisk) that is likely to be *E. coli* topoisomerase III.

Edge Length	13 bp	14 bp	15 bp	16 bp	17 bp	18 bp	19 bp
**Helix turning**	425.5°	458.2°	490.9°	523.6°	556.4°	589.1°	621.8°
**2α**	107.7°	110.3°	112.7°	115.0°	117.2°	119.3°	121.2°
**Geometric turning (-)**	467.7°	470.3°	472.7°	475.0°	477.2°	479.3°	481.2°
**Strain (-)**	0.099	0.026	-0.037	-0.093	-0.142	-0.186	-0.226
**Geometric turning (+)**	612.4°	609.7°	607.3°	605.0°	602.8°	600.7°	598.8°
**Strain (+)**	0.439	0.331	0.237	0.155	0.083	0.020	-0.037
**Handedness**	-	-	-	-	+	+	+

Supplementary Table 1 | Strain analysis of the tensegrity triangles containing the RNA-DNA hybrid 4WJs. 3D geometry is here derived for the hybrid tensegrity triangles with edge length ranging from 13 to 19 bp based on the method in previous studies[1,5]. 2α is the angle between the lines which are perpendicular to the axis of any helix and pass through the tangent points to other helices. Based on 2α, the geometric turning is calculated for left-handed (-) and right-handed (+) tensegrity triangles: $n \times 360° + 2\alpha$ for the left-handed, and $n \times 360° - 2\alpha$ for the right-handed, where *n* is the integer that makes the geometric turning most close to the helix turning. We assume that the RNA-DNA hybrid duplex is the ideal A-form helix[6], with 11 bp per turn of helix and 0.28 nm of rise per bp. The inter-helix distance is chosen to be 2.1 nm. Relatively high strain is marked in red and relatively low strain in green. 14-bp-edged tensegrity triangle has the minimum strain for the left-handed triangle, and therefore is used to generate the negative nodes in our research. For the positive nodes, we chose the 17-bp-edged triangle, but

not the 18-bp-edged one of minimum strain. This is because our previous research indicated that the right-handed triangle with edges 1 bp shorter than the minimum-strain triangle can help increase the yield of topological construction due to the better flexibility of the single-stranded linkers[1].

Strand Name	Sequence
**TKJ-Sca-1.TMP**	TTCTAATACGACTCACTATA**G**GATACAACAGATCCCTGATGAGTCCGTGAGGACGAAA<br>CGAGCTAGCTCGTCGGATCTGttgtatttatCTGAAGCCTGGACGATGCGAACGCCTGATT<br>CCCattatcttgttAGCTAGACTCTACTGGGCCGGCATGGTCCCAGCCTCCTCGCTGGCGC<br>CGGCTGGGCAACATGCTTCGGCATGGCGAATGGGAC
**TKJ-Sca-2.TMP**	TTCTAATACGACTCACTATA**G**GATACAATCGAACCCTGATGAGTCCGTGAGGACGAAA<br>CGAGCTAGCTCGTCGGTTCGAttgttattctGCTACTGGACAACTGCGCTTCCTGGACTAG<br>ACGttctattgtatGACTTCAGCATCATGGGCCGGCATGGTCCCAGCCTCCTCGCTGGCGC<br>CGGCTGGGCAACATGCTTCGGCATGGCGAATGGGAC
**TKJ-Sca-3.TMP**	TTCTAATACGACTCACTATA**G**GATCAAACAGTCGCCTGATGAGTCCGTGAGGACGAAA<br>CGAGCTAGCTCGTCGCGACTGtttgtttattATGCTCTGGACTTGTGTTTCTAACCCTGGAG<br>GATttcttgttatCTGACATCGTACTCGGGCCGGCATGGTCCCAGCCTCCTCGCTGGCGCC<br>GGCTGGGCAACATGCTTCGGCATGGCGAATGGGAC
**TKJ-Staple-1**	GGGAATCACCAGAGCA
**TKJ-Staple-2**	ATCCTCCACCAGTAGC
**TKJ-Staple-P3**	CGTCTAGTGGCTTCAG
**TKJ-Staple-P4**	CATCGTCCACCAGGAAGCGCAGTTGTGGGTTAGAAACACAAGTGGCGTTCG
**TKJ-Staple-N5**	CGTCTAGTCCACCAGGCTTCAG
**TKJ-Staple-N6**	GCATCGTGGAAGCGCAGTTGTGGGTTAGCACAAGTGGCGTTC
**TKJ-Splint-1**	CAGATCC CGAGTACGATGTCAG
**TKJ-Splint-2**	TCGAACCCAGTAGAGTCTAGCT
**TKJ-Splint-3**	CAGTCGCCATGATGCTGAAGTC

Supplementary Table 2 | Sequences of strands used to create the trefoil knots using the junction-based method. All the sequences are written from 5' to 3' end. The coloring has been kept constant with the schematic illustrations in this paper. The sequences suffixed with '.TMP' are the template sequence (after PCR amplification) for producing the RNA scaffolds. The G in bold is the +1 position of transcription. The underlined regions are the regions binding to the primers in the PCR amplification and the regions in red corresponding to the ribozyme sequences that are self-cleaved. Herein, the sequences in blue are actually the resulted RNA sequences after T's are replaced by U's. The sequences shown in lower case are the single-stranded linkers. Unlike the case for DNA, where this region is poly(dT), we avoid the use of long tracts of U's to prevent the slipping of the transcriptase. Note that TKJ-Staple-1 and 2 are used in both staple sets to construct 4WJs with different handednesses. The positive set also includes the TKJ-Staple-P3 and P4, and the negative set also includes the TKJ -Staple-N5 and N6.

Strand Name	Sequence
HBR-Sca-B.TMP	TTCTAATACGACTCACTATA**G**GATAAGTGTCAGTCCTGATGAGTCCGTGAGGACGAAA CGAGCTAGCTCGTCGACTGACACttgttcttatttatttATGCTCCCTGTACTGCGCTTGGACT CTTGCttctattgttattcttCTGTCACCTGTACCTCAGCTACTGGGCCGGCATGGTCCCAGCC TCCTCGCTGGCGCCGGCTGGGCAACATGCTTCGGCATGGCGAATGGGAC
HBR-Sca-R	/Phos/ATCTGTGGACACTAGGttttttttttttttttttttCGTCGGCCTGTCGTGCTGATGGACT AGACGttttttttttttttttttttCTTAGACCTGGAGGATC
HBR-Sca-G1	/Phos/CTCTCGAtttttttCTGAAGCCTGCTATGCGAACGGACATTCCCttttttCATGCAC
HBR-Sca-G2	/Phos/CTCAAGTttttttGATCTACCTGCTTACTCTGTTACGGACTCAGTGttttttAGCTCTT
HBR-Staple-M1	GCAAGAGTGGCCGACG
HBR-Staple-M2	CGTCTAGTGGCTTCAG
HBR-Staple-M3	GGGAATGTGGGAGCAT
HBR-Staple-M4	CATAGCACCATCAGCACGACACCAAGCGCAGTACACCGTTCG
HBR-Staple-P5	GTGTCAGTGGTCTAAG
HBR-Staple-P6	CCTAGTGTGGTAGATC
HBR-Staple-P7	CACTGAGTGGTGACAG
HBR-Staple-P8	GAGTAAGCACCACAGATGATCCTCCACCAGTAGCTGAGGTACACCGTAACA
HBR-Splint-G1	ACTTGAGGTGCATG
HBR-Splint-G2	TCGAGAGAAGAGCT
HBR-Splint-B	AAGTGTCAGTCCAGTAGCTGAG
HBR-Splint-R	CACAGATGATCCTC

Supplementary Table 3 | Sequences of strands used to create the single-stranded RNA-DNA hybrid BR. The strands phosphorylated at 5' end are prefixed by /Phos/. The last two splints are used to circularize the ssRNA blue ring (HBR-Splint-B) and the ssDNA red ring (HBR-Splint-R) before the assembly for the hybrid BR. The resulting blue and red rings are annealed with the two halves of green linear precursor, the two splints for green scaffolds (HBR-Splint-G1 and G2), and all staples to form the assembly complex of hybrid BR. The red ring contains the Nt.AlwI recognition site and the green ring contains the Nt.BspQI recognition site. The splints should be added to form the double-stranded regions for the RNase H and nickase digestions in the cleavage experiment.

Strand Name	Sequence
**HTK-D1**	/Phos/AGTCACTATGTACTTGttttttCGTTCTTAGACCTGGAGGATCATCTGTGGACACT AGGATTCGttttttCTTGTTGATAGCTCATCC
**HTK-D2**	/Phos/CTCTCGATTCTTGTTCttttttCATTCTGTCACCTGTACCTCAGCTACTGGACTGAC ACTCTTCttttttCTTGTTCTTCTAACATCC
**HTK-D3**	/Phos/TCGTCATCTTATCTCGttttttCATTGATCTACCTGCTTACTCTGTTACGGACTCAGT GCTTGCttttttCTTGTATTCATACGATCC
**HTK-Staple-1**	GTGTCAGTGGTCTAAG
**HTK-Staple-2**	CCTAGTGTGGTAGATC
**HTK-Staple-3**	CACTGAGTGGTGACAG
**HTK-Staple-4**	GAGTAAGCACCACAGATGATCCTCCACCAGTAGCTGAGGTACACCGTAACA
**HTK-Splint-1**	AGTGACTGGATCGTAT
**HTK-Splint-2**	TCGAGAGGGATGAGCT
**HTK-Splint-3**	ATGACGAGGATGTTAG
**HTK-R1.TMP**	TTCTAATACGACTCACTATA**G**GATGATAGCTCATCCTGATGAGTCCGTGAGGACGAAAC GAGCTAGCTCGTCGATGAGCTATCAACAAGCGAATCCTAGTGTCCACAGATGATCCTC CAGGTCTAAGAACGCAAGTACATAGTGACTGGGCCGGCATGGTCCCAGCCTCCTCGC TGGCGCCGGCTGGGCAACATGCTTCGGCATGGCGAATGGGAC
**HTK-R2.TMP**	TTCTAATACGACTCACTATA**G**GATCTTCTAACATCCTGATGAGTCCGTGAGGACGAAAC GAGCTAGCTCGTCGATGTTAGAAGAACAAGGAAGAGTGTCAGTCCAGTAGCTGAGGT ACAGGTGACAGAATGGAACAAGAATCGAGAGGGGCCGGCATGGTCCCAGCCTCCTC GCTGGCGCCGGCTGGGCAACATGCTTCGGCATGGCGAATGGGAC
**HTK-R3.TMP**	TTCTAATACGACTCACTATA**G**GATTTCATACGATCCTGATGAGTCCGTGAGGACGAAAC GAGCTAGCTCGTCGATCGTATGAATACAAGGCAAGCACTGAGTCCGTAACAGAGTAA GCAGGTAGATCAATGCGAGATAAGATGACGAGGGCCGGCATGGTCCCAGCCTCCTCG CTGGCGCCGGCTGGGCAACATGCTTCGGCATGGCGAATGGGAC

Supplementary Table 4 | Sequences of strands used to create the double-stranded RNA-DNA hybrid knot. The ssDNA knotted template is prepared using the scaffolds (HTK-D1 to 3), staples (HTK-Staple-1 to 4) and splints (HTK-Splint-1 to 3). The purified complementary RNA strands from the DNA templates (HTK-R1.TMP to 3.TMP) then are annealed with the PAGE-purified ssDNA knotted template and the dsDNA knot is obtained after ligation. The dsDNA circle reference is prepared with similar methods using ssDNA circular template, which is prepared without staples.

Strand Name	Sequence
TKH-Sca.TMP	TTCTAATACGACTCACTATAGGATAAGTGTCAGTCCTGATGAGTCCGTGAGGACGAAACGAGCTAGCTCGTCGACTGACACTTTCTTTCAGGTCCAGTCTTATTCTTGTTTGTCAGACGGATCTTTCTTTATTTCGACTGGACCTGATTTGTTTCTTTCGATCCGTCTGACTTTCCTCAGCTACTGGGCCGGCATGGTCCCAGCCTCCTCGCTGGCGCCGGCTGGGCAACATGCTTCGGCATGGCGAATGGGAC
TKH-Splint-TK	GTGTCAGTCCAGTAGCTG
TKH-Splint-C	GACCTGAAAGAAAGTGTCAGTCCAGTAGCTGAGGAAAGTCAGAC
TKH-Splint-GK	AAGTGTCAGTCCAGTAGCTGAG
TKH-Splint-C2	GACTGGACCTGAAAGAAAGTGTCAGTCCAGTAGCTGAGGAAAGTCAGACGGATC
TKH-Block-C	TCAGGTCCAGTCGAAATAAAGAAAGATCCGTCTGAC

Supplementary Table 5 | Sequences of strands used to create the helix-based topological structures. TKH-Sca.TMP is the DNA template for generating the RNA precursor, which is also the linear species, $\mathbf{L_h}$. The sequences are colored with different colors in the DNA template or assisting DNA splints or block strand (see also **Supplementary Figure 5**). The sequences in italic (colored in red or blue) are designed to form the intrastrand helices for the generation of nodes.

Strand Name	Sequence
Primer1	CAGTCGCCATGATGCTGAAGTC
Primer2	CGTACTCGGGATCTGTTGTATTTATC

Supplementary Table 6 | Sequences of primers in the RT-PCR. Primer1 is the primer for RT and therefore also the reverse primer for the subsequent PCR. In fact, it is the same sequence as TKJ-Splint-3 in **Supplementary Table 2**. Primer2 is the forward primer for the RT-PCR.

Supplementary References

1. Liu, D., Chen, G., Akhter, U., Cronin, T. M. & Weizmann, Y. Creating complex molecular topologies by configuring DNA four-way junctions. *Nature Chem.* 2016, **8**(10): 907-914.
2. Wang, H., Di Gate, R. J. & Seeman, N. C. An RNA topoisomerase. *Proc. Natl. Acad. Sci. USA* 1996, **93**(18): 9477-9482.
3. Mueller, J. E., Du, S. M. & Seeman, N. C. Design and synthesis of a knot from single-stranded DNA. *J. Am. Chem. Soc.* 1991, **113**(16): 6306-6308.
4. Rodbard, D. & Chrambach, A. Estimation of molecular radius, free mobility, and valence using polyacrylamide gel electrophoresis. *Anal. Biochem.* 1971, **40**(1): 95-134.
5. Birac, J. J., Sherman, W. B., Kopatsch, J., Constantinou, P. E. & Seeman, N. C. Architecture with GIDEON, a program for design in structural DNA nanotechnology. *J. Mol. Graph. Model.* 2006, **25**(4): 470-480.
6. Egli, M., Usman, N., Zhang, S. G. & Rich, A. Crystal structure of an Okazaki fragment at 2-A resolution. *Proc. Natl. Acad. Sci. USA* 1992, **89**(2): 534-538.
